# Intraspecific Inversions Pose a Challenge for the *trnH-psbA* Plant DNA Barcode

**DOI:** 10.1371/journal.pone.0011533

**Published:** 2010-07-13

**Authors:** Barbara A. Whitlock, Amanda M. Hale, Paul A. Groff

**Affiliations:** 1 Department of Biology, University of Miami, Coral Gables, Florida, United States of America; 2 Department of Biological Sciences, Texas Christian University, Fort Worth, Texas, United States of America; Montreal Botanical Garden, Canada

## Abstract

**Background:**

The chloroplast *trnH-psbA* spacer region has been proposed as a prime candidate for use in DNA barcoding of plants because of its high substitution rate. However, frequent inversions associated with palindromic sequences within this region have been found in multiple lineages of Angiosperms and may complicate its use as a barcode, especially if they occur within species.

**Methodology/Principal Findings:**

Here, we evaluate the implications of intraspecific inversions in the *trnH-psbA* region for DNA barcoding efforts. We report polymorphic inversions within six species of Gentianaceae, all narrowly circumscribed morphologically: *Gentiana algida, Gentiana fremontii, Gentianopsis crinita, Gentianopsis thermalis, Gentianopsis macrantha* and *Frasera speciosa*. We analyze these sequences together with those from 15 other species of Gentianaceae and show that typical simple methods of sequence alignment can lead to misassignment of conspecifics and incorrect assessment of relationships.

**Conclusions/Significance:**

Frequent inversions in the *trnH-psbA* region, if not recognized and aligned appropriately, may lead to large overestimates of the number of substitution events separating closely related lineages and to uniting more distantly related taxa that share the same form of the inversion. Thus, alignment of the *trnH-psbA* spacer region will need careful attention if it is used as a marker for DNA barcoding.

## Introduction

DNA barcoding, the use of short, standardized orthologous DNA sequences to identify species, promises a rapid and efficient method to explore the dimensions of biodiversity. The mitochondrial CO1 gene appears to have wide utility in discriminating among animal lineages [1], [2], but a similar general barcode for plants has remained elusive [3]. The chloroplast *trnH-psbA* spacer has been proposed as one such barcode for plants, either alone or in conjunction with other sequences [4]–[6]. Recently, the Consortium for the Barcode of Life (CBOL) Plant Working Group [2], [7] proposed two other chloroplast regions, the protein-coding *rbcL* and *matK,* as a 2-locus combination barcode. However, the Consortium recognized that these two genes may need to be supplemented by additional loci to discriminate among closely related species; the *trnH-psbA* region remains the leading candidate as a source of additional data [2], [7], [8]. Here, we explore a complication of using *trnH-psbA* that has been overlooked in the plant barcode literature: frequent inversions in a region of *trnH-psbA* that is flanked by inverted repeats. Although inversions in *trnH-psbA* have been noted previously [e.g., [9],[10]], we report multiple examples of *intra*specific inversions in this region, in six species of Gentianaceae. Since barcoding efforts focus on identification of species, we hypothesize that such intraspecific polymorphisms will be especially problematic for this research program, if the inversions are not detected and accommodated appropriately in alignments.

In many plant lineages, the *trnH-psbA* region shows many of the features deemed desirable in a barcode, including short length (often <500bp), suspected ubiquity in plants, high interspecific sequence divergence, and universal flanking primers that allow for easy amplification and sequencing from both high molecular weight and degraded DNA [4], [11]–[14]. However, in some plant lineages, *trnH-psbA* does not amplify well, or amplifies as multiple bands [15]–[16]. It is occasionally longer than is currently feasible for a barcode [2], [12], [17], may have mononucleotide repeats that are difficult to sequence accurately [2], [16], [18]–[20] and insertion events, including insertions of other genes [21] into the region. Within some groups, *trnH-psbA* is not sufficiently variable to distinguish among closely related species [e.g. [15],[20]] and in others intraspecific variation is high [22].

In our studies within Gentianaceae, *trnH-psbA* is often easy to amplify and sequence, even from degraded samples, and shows high levels of interspecific and intraspecific sequence variation. However, one source of intraspecific variation that may prove problematic for DNA barcoding is the presence of different configurations of a 25–27bp inversion associated with inverted repeats. Here we report six examples of such inversions within species of Gentianaceae that are narrowly defined morphologically. This intraspecific variation, combined with the generally short length of the *trnH-psbA* region, suggests that typical methods of alignment of these sequences could result in misassignment of conspecific sequences that show different forms of the inversion.

In a paper exploring the phylogenetic utility of *trnH-psbA*, Sang et al. [9] identified inversions within this region that they interpreted as highly homoplasious within the genus *Paeonia*. Other studies have found small inversions in several other non-coding chloroplast sequences, usually associated with flanking inverted repeats, or palindromic sequences [23]–[27]. Together, the flanking inverted repeats (or stems) and intervening inversion-prone regions (or loops) suggest a hairpin structure that may play a role in the stability of mRNA [28]–[30]. These inversions appear to be common among non-coding chloroplast regions [29], the same regions that show high substitution rates desirable in a DNA barcode.

Here, we explore potential effects of intraspecific inversions in *trnH-psbA* on the ability of this marker to identify species. We align and analyze sequences from six polymorphic species together with sequences from 15 other species of Gentianaceae. We use sets of analyses, employing different methods typically used in barcoding, on two alternative alignments of the inversion region. In the first, we use unaltered (“raw”) sequence data in which different taxa exhibit either one or the other of the two different inversion configurations. In the second, we invert the sequence of the loop region for 19 taxa, by removing and replacing one form of the inversion with the reverse complement of its sequence, to maximize sequence homology across the inversion region. By analyzing the phylogenetic trees and sequence divergences of these two alternative alignments, we test the hypothesis that distantly related sequences with the same configuration of the inversion may cluster more closely than conspecific sequences with different forms of the inversion. This result would indicate a risk of incorrect species identification when *trnH-psbA* is employed in DNA barcoding.

## Materials and Methods

### Taxon sampling

Our dataset began with 3–4 *trnH-psbA* sequences from each of six species of Gentianaceae, primarily North American, that were found to be polymorphic for the inversion region. These include two species from subtribe Gentianinae (*Gentiana algida* and *Gentiana fremontii* representing two distant sections of *Gentiana*) and four from subtribe Swertiinae (*Gentianopsis thermalis, Gentianopsis macrantha, Gentianopsis crinita*, and *Frasera speciosa*). We aligned these sequences with sequences from 15 other North American species of Gentianaceae that were chosen specifically to reflect different scenarios that may be encountered in barcoding studies. Our final dataset thus included some taxa with very dense sampling (e.g., very closely related North American species of *Gentianopsis*) and some taxa with sparse sampling (e.g., *Gentiana* sect. *Frigidae* represented by only one species, *G. algida*). In this manner, we hoped to reveal situations in which intraspecific inversions may cause problems for DNA barcoding, rather than to test the monophyly of species that are polymorphic for the inversion region. Voucher information, as well as the configuration seen in the inversion region, is given in Table S1.

### Molecular methods

Total genomic DNA was extracted from leaf material that was dried in silica gel or from herbarium specimens, using a 6% PVP method [31]. We sequenced the *trnH-psbA* spacer using primers trnHf [32] and psbA3'f [9]. PCR products were cleaned with a standard exo-SAP procedure. Double-stranded products were sequenced in both directions using ABI BigDye dye-terminators and cycle-sequencing protocols. Sequencing reactions were run on an ABI 3730xl DNA analyzer. Sequences were assembled using Sequencher 3.0 (GeneCodes Corp., Ann Arbor, Michigan). All sequences are available in GenBank (accession numbers HM460843-HM460877).

### Alignment

Before alignment, primer sequences were removed from both ends of the sequences, so that all sequences begin and end at homologous sites. We then constructed two data matrices on which to perform alignments: Matrix 1 in which all sequences reflecting the raw sequence data and exhibiting two different forms of the inversion region (the “raw sequence” matrix); and Matrix 2 with the inversion replaced with the reverse complement of its sequence for 19 sequences, such that sequence homology was maximized across the inversion region (the “uniform inversion” matrix).

Both matrices were aligned using default settings in ClustalX [33]. Subsequent analyses were performed on the direct output of ClustalX, to emulate the automated process that has been proposed for barcoding (“unedited” matrices). However, because of length variation within *trnH-psbA*, sequences were poorly aligned across species in the 5′ half. We subsequently manually edited the alignments by realigning the first 45 bp of short sequences (“edited” matrices).

### Analyses

We performed neighbor-joining (NJ), UPGMA, and maximum parsimony (MP) analyses on all matrices, following previously published barcoding studies [e.g., [34]], using PAUP* [35]. Parsimony analyses used TBR, a single sequence additional replicate, and were limited to 5000 trees. Although these methods would not generally be considered robust for phylogenetic analyses, advocates of barcoding emphasize species identification rather than robust inference of phylogenetic relationships [3], [13].

Following previous barcoding studies, the number of variable sites within species, maximum intraspecific uncorrected p-distance, and minimum interspecific uncorrected p-distance were calculated for the six species that are polymorphic for the inversion region, using alignments based on both the raw sequence matrix and the uniform inversion matrix. The minimum interspecific distances were calculated by comparing sequences of the species of interest to sequences of all remaining species in the dataset. Characters that include insertions and deletions for any of the conspecific sequences were noted but excluded from these calculations.

## Results

**Figure 1:**
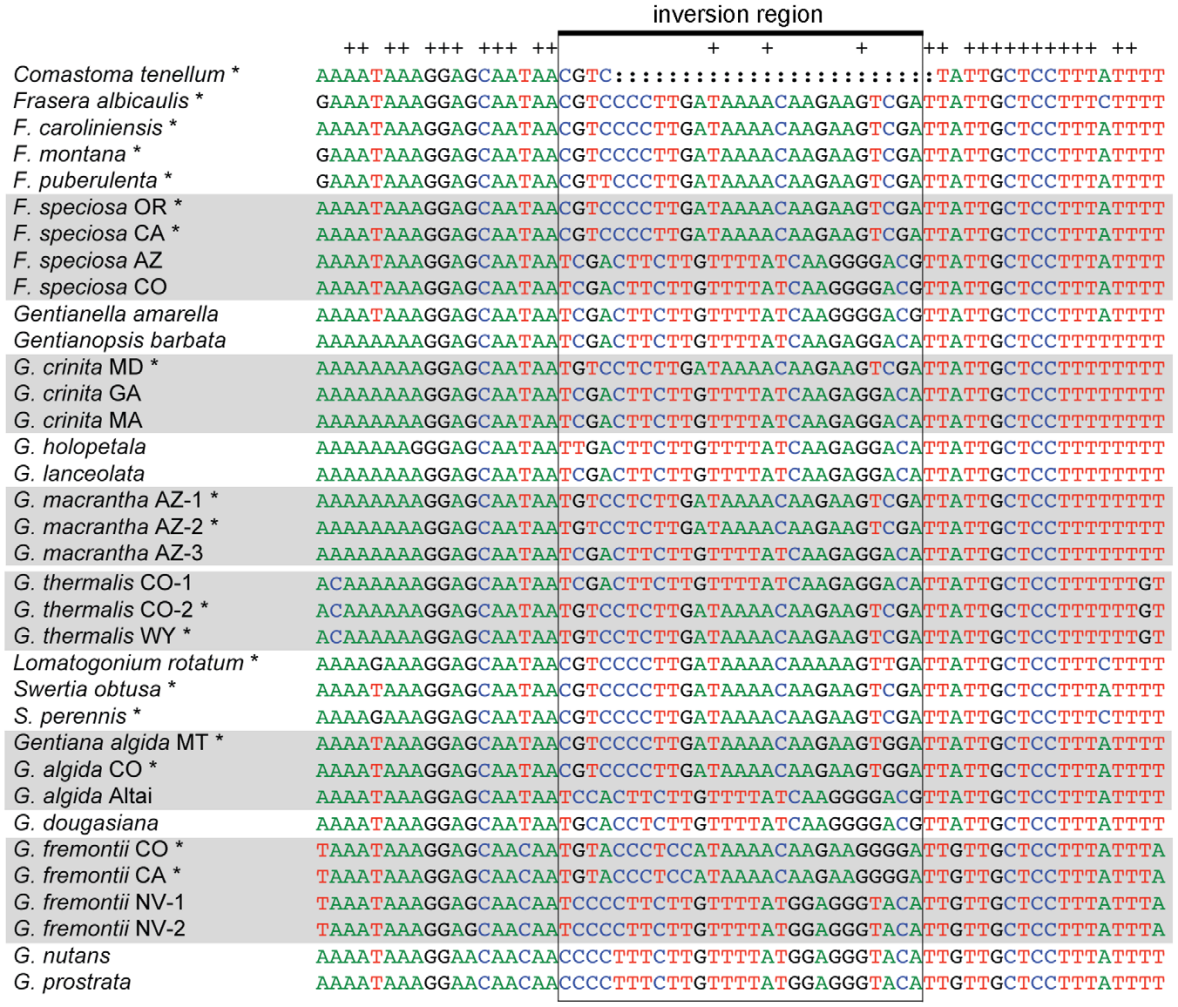
Portion of 35 *trnH-psbA* sequences including 25–27bp inversion and 18bp flanking inverted repeats. Groups of conspecific sequences are shaded. Asterisks mark sequences with the A inversion configuration. Plus signs at the top of the alignment mark invariant sites. Inversion region is in box.

The *trnH-psbA* region amplified easily from all samples included in this study. Lengths of sequences range from 214 (*Gentiana douglasiana*) to 489 (*Frasera puberulenta*). In most of the taxa included, the loop region is at least 27bp (Figure 1). In *Gentianopsis*, the loop region is at least 25bp (Figure 1). The sequence of *Comastoma tenellum* has a large deletion encompassing all but 3 bp of the loop region and 1bp of the flanking region that appears to have occurred after the most recent inversion event in that lineage (Figure 1). The inversion region of all taxa sampled is flanked on both sides by a minimum of 18 bp of reverse complementary, or palindromic, sequences (Figure 1).

Of the 35 sequences included in the matrix, 19 have one easily identifiable configuration of the inversion region, here designated the A form, and 16 have the reverse complement, or the B form (Figure 1, Table S1). Additional sampling of taxa, plus a more robust phylogeny of temperate gentians, is needed to infer which form of the inversion is plesiomorphic within Gentianaceae. We arbitrarily chose sequences with the A form, and replaced their inversion region with the reverse complementary sequence to maximize homology with the B form. Because of high sequence conservation within the inversion region (Figure 1), this process was straightforward.

ClustalX resulted in alignments for both matrices that appeared less than ideal for phylogenetic analyses. The short lengths of sequences of four species of *Gentiana* (*G. fremontii, G. prostrata, G. nutans,* and *G. douglasiana*) and two species of *Gentianopsis* (*G. macrantha* and *G. lanceolata*), all less than 300bp, resulted in the misalignment of their first 40–45 bp relative to the remaining sequences, despite high conservation of sequence. We refer to the original ClustalX alignments as “unedited” in the discussion below. All analyses were also performed on “edited” alignments in which the first 40–45bp have been manually adjusted.

**Figure 2:**
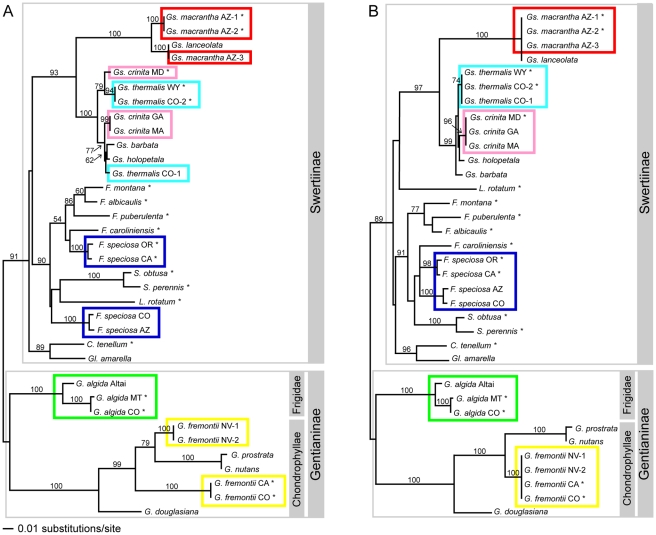
Neighbor-joining trees resulting from two treatments of *trnH-psbA* inversion. (A) NJ tree resulting from analysis of unedited raw sequence matrix, in which different taxa have either one or the other of two inversion configurations, and (B) NJ tree resulting from analysis of unedited uniform inversion matrix, in which the loop region of sequences with the A configuration has been replaced with its reverse complement. Conspecific sequences are indicated in colored boxes. *Gs.*  =  *Gentianopsis, F.*  =  *Frasera, S.*  =  *Swertia, L.*  =  *Lomatogonium*, *C.*  =  *Comastoma, Gl.*  =  *Gentianella*, *G.*  =  *Gentiana*. Two subtribes of Gentianaceae and two sections of *Gentiana* are indicated in gray boxes. Sequences with the A configuration are noted with an asterisk. Bootstrap values >50% are given above branches. The scale bar refers to both trees.

Analyses using different methods (NJ, UPGMA, MP) resulted in different trees for the edited vs. unedited alignments; the trees differed in relationships among individuals, species and genera. In the remaining discussion, we focus primarily on relationships among conspecific sequences and how they differ due to treatment of the inversion region. Only the results of NJ analyses on the unedited alignments are shown in Figure 2 and Table 1; however, these results illustrate common patterns seen in all analyses discussed in more detail in the text below.

**Table 1:** Characteristics of conspecific *trnH-psbA* sequences of Gentianaceae polymorphic for the inversion region^a^.

				Raw sequence matrix			Uniform inversion matrix		
Taxon	N	Sequence length (bp)	Inversion length (bp)	# Variable sites	Maximum intraspecific distance	Minimum interspecific distance	# Variable sites	Maximum intraspecific distance	Minimum interspecific distance
*Frasera speciosa*	4	378−397	27 (6.8−7.1%)	25 (6.8%)	0.06424	0.04852	4 (1.1%)	0.04788	0.04854
*Gentianopsis crinita*	3	444	25 (5.6%)	17 (3.8%)	0.01798	0.01117	0	0.00225	0.01117
*G. macrantha*	3	284	25 (8.8%)	17 (6.0%)	0.02807	0	0	0	0
*G. thermalis*	3	424−425	25 (5.9%)	17 (4.0%)	0.01887	0.00937	0	0	0.00939
*Gentiana algida*	3	343−374	27 (7.2−7.9%)	23 (6.7%)	0.04053	0.1147	2 (0.6%)	0.0114	0.11615
*G. fremontii*	4	226	27 (12%)	21 (9.3−9.7%)	0.09292	0.08445	0	0	0.08445

a Calculations were performed on unedited alignments. Similar results were obtained from manually adjusted, edited alignments. Gapped characters due to insertions and deletions were excluded from calculations. All distances were measured as uncorrected p-distances. Minimum interspecific distances were calculated by comparing the sequences of the species of interest to sequences of all other species in the dataset.

### Raw sequence analyses

All analyses of the raw sequence matrices, with both edited and unedited alignments, resulted in trees showing deep relationships consistent with our understanding of the phylogeny of these taxa [36](Figure 2A). In all trees (NJ, UPGMA, MP), sequences from the six genera from subtribe Swertiinae consistently formed a group and the remaining sequences from *Gentiana*, placed in subtribe Gentianinae, formed a second group. Within *Gentiana,* the species corresponding to *Gentiana* section *Chondrophyllae* consistently clustered together. Within Swertiinae, the sequences of *Gentianopsis* clustered together.

In five of the six species polymorphic for the inversion region, conspecific sequences did not cluster together in any analysis of the raw sequence matrix (e.g., Figure 2A). Within *Gentianopsis*, one of the three sequences of *G. crinita* consistently clustered with two of the four sequences of *G. thermalis* (all with the A form of the inversion region), and one of the three sequences of *G. macrantha* consistently appeared more closely related to *G. lanceolata* (both with the B form of the inversion). Within *Gentiana*, sequences of *G. fremontii* with the B form always appeared more closely related to sequences from *G. prostrata* and *G. nutans*, also with the B form, than with conspecific sequences.

In all analyses of the raw sequence matrices, the four sequences of *Frasera speciosa* never clustered together; however, relationships of the sequences with the two conformations varied in the results of the different analyses. The two sequences with the A form of the inversion either clustered with sequences from congeners *F. puberulenta, F. albicaulis*, and *F. montana* (NJ and UPGMA trees; Figure 2A), or formed a polytomy with the other species of *Frasera* plus sequences of *Lomatogonium* and *Swertia* (MP trees). Relationships of *F. speciosa* sequences with the B form of the inversion also varied, clustering with *Lomatogonium* and *Swertia* (NJ trees), forming a basal lineage of a combined *Frasera* group (UPGMA trees), or forming a basal lineage of a combined clade including other *Frasera, Lomatogonium* and *Swertia* (MP trees).

In contrast to the five other species, the three sequences of *Gentiana algida* consistently clustered together in all analyses of the raw sequence matrix despite their polymorphic inversion region. This species is the only representative of *Gentiana* section *Frigidae* in this study.

### Uniform matrix analyses

Analyses of the uniform inversion matrix consistently showed close relationships among conspecific sequences in five of the six species polymorphic for the inversion region (e.g., Figure 2B). In all trees, conspecific sequences of *Gentianopsis crinita, Gentianopsis thermalis, Gentiana algida,* and *Gentiana fremontii*, consistently clustered together, in line with our understanding of species limits within these taxa based on morphology, geography, and other genetic markers. The three *trnH-psbA* sequences of *Gentianopsis macrantha* are identical to each other, once the inversion region of sequences with the A form was replaced with the reverse complements, and also identical to the sequence of *Gentianopsis lanceolata*. Other markers may distinguish these species (Whitlock and Groff, unpubl. data).

In all trees, all sequences of *Frasera* grouped together; however, relationships among the four sequences of *F. speciosa* differed in trees from different analyses. All *F. speciosa* sequences formed a clade in MP trees of the unedited alignment and clustered together in UPGMA trees. In NJ trees, two individuals of *F. speciosa* clustered with the sequence of *F. caroliniensis*. In the MP strict consensus from analysis of the edited alignment, relationships among the *F. speciosa* sequences were unresolved.

Divergence among conspecific sequences was greater in the raw sequence matrix than in the uniform matrix. The number of variable sites within species in *trnH-psbA*, with original configurations of the inversion, ranged from 17 to 25, or 3.8–9.7% of the total length of *trnH-psbA* (Table 1). This is substantially higher than the average sequence divergence (2.7%) found by comparing randomly selected pairs of congeneric sequences [6]. The majority of these sites occurred within the loop region. When the inversion of the A form was replaced with the reverse complementary sequence in the uniform sequence matrix, the number of variable sites within species decreased to 0–4, or 1.1% (Table 1). In three species, *Gentianopsis crinita*, *Gentianopsis macrantha*, and *Gentiana fremontii*, all conspecific sequences were identical, once the inversions were flipped. The three sequences of *Gentianopsis thermalis* were identical with the exception of a 1bp length variant in a mononucleotide repeat. Because of the short lengths of some *trnH-psbA* sequences, the number of variable sites within species in the loop region of the raw sequence matrix, due to false homology assessment, represents a large proportion overall of the sites in this marker.

In both the edited and unedited alignments of the raw sequence matrix, the maximum intraspecific uncorrected p-distances in five of the six polymorphic species are greater than the minimum interspecific distances for these species. In all, the maximum intraspecific distances are between sequences with different conformations of the inversion region, and the minimum interspecific distances occur between sequences with the same conformation. For example, within *Gentianopsis thermalis*, the distance between sequences from Colorado and Wyoming plants with different conformations of the inversion (0.01887) is more than twice the distance between the Wyoming *G. thermalis* and a specimen of *Gentianopsis holopetala* (0.00937)(Table 1). This pattern disappears in the uniform sequence matrices, in which the maximum intraspecific distances are all less than or equal to the minimum interspecific distances. Maximum intraspecific distances are always greater than the minimum interspecific distances for the sixth species, *Gentiana algida.* This is also the only species in which the sequences consistently cluster together in analyses of the raw sequence matrices.

## Discussion

Our data show that intraspecific inversions can lead to an overestimate of divergence among conspecific sequences and misleading estimates of relationships among closely related species. We suspect that comparisons and alignments of sequences with alternate inversion states would compromise other, more sophisticated tree-building and phenetic analyses. Because the 25–27bp region subject to inversion makes up a large proportion (5–11%) of the total length of *trnH-psbA*, DNA barcoding may be more likely to fail in distinguishing among closely related species, if the inversion is not recognized and realigned so that all sequences have the same configuration of the inversion. For example, sequences of *Gentianopsis crinita* and *G. thermalis* with the same configuration of the inversion only differ by five substitutions (excluding indels) that are all located outside the inversion region (Figure 2A). Conversely, conspecific sequences of *G. crinita* with different inversion configurations differ at 17 sites, all within the inversion region. Conspecific sequences with different inversion configurations thus appear more distantly related to each other than sequences from closely related species that share inversion configurations, because of incorrect homology assessment within the inversion region. As comparisons are made with more distantly related taxa, this problem may attenuate. For instance, in our analyses, *trnH-psbA* sequences easily distinguish the genera *Gentiana* and *Gentianopsis,* regardless of the state of the inversion (Figure 2A). If such intraspecific inversions occur more generally, they may prove even more problematic for barcoding than for phylogenetic analyses, particularly if alternate inversion states for each species have not yet been sampled and included in the barcoding profile. Furthermore, this complication will not be mitigated by a two- or multi-locus approach, in which a more conserved coding region is first used to identify an unknown to genus or family [5], [6] if *trnH-psbA* is employed as the more variable marker. One of the appeals of DNA barcoding is its potential to distinguish among closely related species that are morphologically nearly identical, but unrecognized intraspecific inversions may compromise this discriminatory power.

Inversions in the *trnH-psbA* region are not unique to Gentianaceae. *Inter*specific inversions have been identified widely in Angiosperms [10] and are often revealed in phylogenetic studies of closely related species [9], [10], [30], [32], [37]–[41], suggesting inversion events are frequent. Furthermore, small inversions are not limited to the *trnH-psbA* region within the chloroplast genome. They have been found in *rpl16* [24], *psbC-trnS* [27], *trnL-trnF* [29], and *atpB-rbcL* [42] among others.

Some authors [18], [30], [43] have speculated that *intra*specific inversions might be problematic for barcoding, but did not test this hypothesis with empirical data. Prior to this paper, intraspecific inversions have rarely been reported but are not unknown. Two studies have previously documented intraspecific inversions in *trnH-psbA*, including a 30bp inversion in two species of *Silene* [30] and a 6bp inversion within *Magnolia macrophylla* [37]. Furthermore, intraspecific inversions have also been found in the *trnL-trnF* spacer region, another commonly used phylogenetic marker that has also been proposed for DNA barcoding [44], in *Jasminum elegans* [29] and several species of Bryophytes [45]. We suspect that more examples of intraspecific inversions in chloroplast DNA will be found as sampling within species increases, but the present study appears to represent the largest dataset yet available of intraspecific *trnH-psbA* inversions within a plant family.

Although many species of Gentianaceae are conspicuous and well-known wildflowers, some are cryptic, especially when vegetative. Even when flowering, species of *Gentianopsis* (“fringed gentians”) have proven challenging to distinguish morphologically, as shown by the frequent misidentifications in herbaria and databases. Thus, the concept of using DNA sequence data to identify species is appealing. We have successfully employed *trnH-psbA* and other markers to identify tiny rosettes, lacking key floral characters, to species (unpubl. data). However, our ability to do so rests on pre-existing densely-sampled phylogenies that allowed us to identify lineages. These in turn rely on our taxonomic and morphological expertise that enabled us to infer how lineages correspond to species limits. Our strategy of sampling within species to clarify intra- and interspecific variation led to the discovery of intraspecific inversions in *trnH-psbA*.

The suggestion that DNA barcoding could be performed by non-professionals, or automated [e.g., [46]], is appealing, but may be premature given typical current practice. Widely used alignment programs such as ClustalX do not screen for inversions. We detected inversions in the data presented here by a labor-intensive visual inspection of alignments. A recent review of non-coding chloroplast DNA [43] similarly concluded that detection of inversions depends on taxonomic sampling as well as the experience of the researchers. Thus advocates of DNA barcoding may want to avoid markers such as non-coding chloroplast sequences whose alignment requires close scrutiny for structural changes. However, there may be potential to automate this scrutiny. Software packages and online resources already exist, (e.g., EMBOSS [47]) that can be used to identify palindromic regions that often flank sequences prone to inversion. Our data for *trnH-psbA* may serve as a useful caution: algorithms that screen for structural mutations, including inversions as well as insertions and deletions, may need to be incorporated into the bioinformatic toolkit to be used in DNA barcoding generally, for all markers.

Sequence regions that exhibit inversions are often excluded from phylogenetic analyses because they appear too homoplasious [e.g., [9],[39]]. However, these inversions may provide valuable information of relationships among populations, and may provide evidence for the presence of cryptic species. In our analyses, two individuals of *Frasera speciosa* that share the same inversion form also share two unique substitutions and two indels, of 9bp and 26bp, suggesting that their shared configuration of the inversion may reflect shared history. Omitting regions subject to inversion from analyses may also result in the loss of informative substitutions within the inversion region that may serve to distinguish among closely related species (Figure 1). In phylogenetic analyses, it may be best practice to identify inversion regions, replace one inversion configuration with its reverse complement to maximize homology, and code the inversion as a single binary character, comparable to an indel. These subtleties in assessing the information content of sequence data suggest that there will always be tension between our desire to automate species identification and our need for informed human judgment as an input into the process. Barcodes for species identification, like barcodes we encounter at the cashier, may have the potential to go infuriatingly wrong. It is up to us to regulate and fine-tune the technology, and then employ it in a way that will truly meet our needs.

## Supporting Information

Table S1 Specimens included in analyses, their voucher information, the configuration seen in the inversion region, and GenBank accession numbers. Sequences from conspecific specimens are differentiated in the Figures by an abbreviation of the locality where they were collected, shown parenthetically after the taxon name below.
(0.06 MB DOC)
