# Supplementary material for: Intraspecific Inversions Pose a Challenge for the trnH-psbA Plant DNA Barcode
Source: PLoS One. 2010 Jul 13;5(7):e11533. doi: 10.1371/journal.pone.0011533 (PMC2903610; doi:10.1371/journal.pone.0011533)
Supplement: Table S1 — Specimens included in analyses, their voucher information, the configuration seen in the inversion region, and GenBank accession numbers. Sequences from conspecific specimens are differentiated in the Figures by an abbreviation of the locality where they were collected, shown parenthetically after the taxon name below. (0.06 MB DOC) [file pone.0011533.s001.doc]

| **Taxa** | **Voucher information** | **Inversion configuration** | **GenBank Accession Numbers** |
| --- | --- | --- | --- |
| Swertiinae |  |  |  |
| *Comastoma tenellum* (Rottb.) Toyokuni | Groff 07-OR-5-A.2 | A form | HM460843 |
| *Frasera albicaulis* Douglas ex. Griseb. | Groff 07-NV-2-A.1 | A form | HM460844 |
| *F. caroliniensis* Walter | Groff 09-GA-1-B.1 | A form | HM460845 |
| *F. montana* Mulford | Groff 08-ID-12-A.1 | A form | HM460846 |
| *F. puberulenta* Davidson | Groff 08-CA-4-A.1 | A form | HM460847 |
| *F. speciosa* Douglas ex Griseb. (OR) | Groff 07-OR-4-A.1 | A form | HM460848 |
| *F. speciosa* Douglas ex Griseb. (CA) | Groff 08-CA-5-A.1 | A form | HM460849 |
| *F. speciosa* Douglas ex Griseb. (AZ) | Groff 07-AZ-12-A.1 | B form | HM460850 |
| *F. speciosa* Douglas ex Griseb. (CO) | Groff 08-CO-6-A.1 | B form | HM460851 |
| *Gentianella amarella* (L.) Böerner | Groff, Whitlock, and Eliason 07-CA-1-A | B form | HM460852 |
| *Gentianopsis barbata* (Froel.) Ma | Ho et al. 321 (GH) | B form | HM460853 |
| *G. crinita* (Froel.) Ma (MD) | Hill 12423 (GH) | A form | HM460854 |
| *G. crinita* (Froel.) Ma (GA) | T. Patrick s.n. GA | B form | HM460855 |
| *G. crinita* (Froel.) Ma (MA) | Groff and Whitlock s.n. MA | B form | HM460856 |
| *G. holopetala* (A. Gray) Iltis | Groff and Whitlock 06-2-A | B form | HM460857 |
| *G. lanceolata* (Benth.) Iltis | Iltis et al. 1044 (WIS) | B form | HM460858 |
| *G. macrantha* (D.Don ex G.Don) Iltis (AZ-1) | Groff 07-AZ-14-A.1a | A form | HM460859 |
| *G. macrantha* (D.Don ex G.Don) Iltis (AZ-2) | Groff 07-AZ-15-A.1 | A form | HM460860 |
| *G. macrantha* (D.Don ex G.Don) Iltis (AZ-3) | Mason and Mason 3984 (NY) | B form | HM460861 |
| *G. thermalis* (Kuntze) Iltis (CO-1) | Groff 08-CO-114-A.1 | B form | HM460862 |
| *G. thermalis* (Kuntze) Iltis (CO-2) | Groff 08-CO-155-A.1 | A form | HM460863 |
| *G. thermalis* (Kuntze) Iltis (WY) | Groff 08-WY-7-A.1 | A form | HM460864 |
| *Lomatogonium rotatum* (L.) Fr. ex Fernald | Groff 08-UT-212-A.1 | A form | HM460865 |
| *Swertia obtusa* Ledeb. | Gemeinholzer et al. 3886 (B) | A form | HM460866 |
| *S. perennis* L*.* | Groff 08-CO-142-A.2 | A form | HM460867 |
| Gentianinae |  |  |  |
| *Gentiana algida* Pall. (MT) | Groff 08-MT-14-A.1 | A form | HM460868 |
| *G. algida* Pall. (CO) | Groff 08-CO-108-A.1 | A form | HM460869 |
| *G. algida* Pall. (Altai) | Gemeinholzer et al. 212/07 (B) | B form | HM460870 |
| *G. douglasiana* Bong. | Parker 5147 (ALA) | B form | HM460871 |
| *G. fremontii* Torr. (CO) | Groff 08-CO-3-A.1 | A form | HM460872 |
| *G. fremontii* Torr. (CA) | Groff 07-CA-10-A.1 | A form | HM460873 |
| *G. fremontii* Torr. (NV-1) | Reveal 631 (NY) | B form | HM460874 |
| *G. fremontii* Torr. (NV-2) | Maguire and Holmgren 25803 (WTU) | B form | HM460875 |
| *G. nutans* Bunge | Hartman and Nelson 22157 (NY) | B form | HM460876 |
| *G. prostrata* Haenke | Groff 07-OR-1-A.1 | B form | HM460877 |
